# *Giardia* telomeres and telomerase

**DOI:** 10.1007/s00436-024-08200-6

**Published:** 2024-04-08

**Authors:** Francisco Alejandro Lagunas-Rangel

**Affiliations:** 1 Department of Surgical Sciences, Uppsala University, Husargatan 3, BMC Box 593, 751 24 Uppsala, Sweden; 2 Department of Genetics and Molecular Biology, Centro de Investigación y de Estudios Avanzados del Instituto Politécnico Nacional, Av Instituto Politécnico Nacional 2508, San Pedro Zacatenco, Gustavo A. Madero, 07360 Mexico City, Mexico

**Keywords:** Intestinal parasite, Chromosome ends, TERT, Telomerase RNA, Telomere shortening

## Abstract

*Giardia duodenalis*, the protozoan responsible for giardiasis, is a significant contributor to millions of diarrheal diseases worldwide. Despite the availability of treatments for this parasitic infection, therapeutic failures are alarmingly frequent. Thus, there is a clear need to identify new therapeutic targets. *Giardia* telomeres were previously identified, but our understanding of these structures and the critical role played by *Giardia* telomerase in maintaining genomic stability and its influence on cellular processes remains limited. In this regard, it is known that all *Giardia* chromosomes are capped by small telomeres, organized and protected by specific proteins that regulate their functions. To counteract natural telomere shortening and maintain high proliferation, *Giardia* exhibits constant telomerase activity and employs additional mechanisms, such as the formation of G-quadruplex structures and the involvement of transposable elements linked to telomeric repeats. Thus, this study aims to address the existing knowledge gap by compiling the available information (until 2023) about *Giardia* telomeres and telomerase, focusing on highlighting the distinctive features within this parasite. Furthermore, the potential feasibility of targeting *Giardia* telomeres and/or telomerase as an innovative therapeutic strategy is discussed.

## Introduction

*Giardia duodenalis*, the causative agent of giardiasis, is a microaerophilic, flagellate, binucleate protozoan responsible for more than 300 million cases of diarrheal disease worldwide. Notably, its impact is especially pronounced in developing and low-income countries (Cernikova et al. 2018). Classified within the order Diplomonadida, *Giardia* belongs to the Metamonada group within the Excavata supergroup (Burki et al. 2020). A distinctive feature of *Giardia* is its highly compact genome (12.6 Mb) (Xu et al. 2020), accompanied by a significant reduction of several cellular processes components. More specifically, it lacks certain organelles common in other eukaryotic cells, such as the conventional Golgi apparatus, peroxisomes, and respiratory mitochondria (Cernikova et al. 2018).

*Giardia* undergoes a dynamic life cycle, characterized by two distinct phases: the motile, vegetative trophozoite form and the resistant, highly infective cyst form (Einarsson et al. 2016a). After ingestion by the host, the *Giardia* cyst begins its activation within the gastrointestinal tract, responding to acidic conditions in the stomach and subsequently encountering bile and trypsin in the duodenum. This activation signals the beginning of the release of motile trophozoites in the proximal part of the small intestine. Trophozoites, thriving in the nutrient-rich but oxygen-deprived upper intestinal tract, undergo a phase of strong proliferation. They take advantage of their adhesive discs to anchor themselves firmly to the intestinal villi and thus skillfully resist peristalsis. As parasite density increases, trophozoites move into the lower intestinal tract, where they encounter a dynamic series of environmental changes. These include altered cholesterol levels, elevated pH, and increased bile and lactic acid concentrations. Fascinatingly, in the midst of these altered conditions, a subset of trophozoites undergo a transformative change, ultimately giving rise to infective cysts. These cysts are eventually excreted in the feces, ready to initiate new infections (Barash et al. 2017; Lagunas-Rangel et al. 2021).

Although *Giardia* infections can sometimes go unnoticed, sometimes, the symptoms of giardiasis may include more than just watery diarrhea. Patients may experience nausea, epigastric pain, and in some cases, weight loss (Einarsson et al. 2016a). Of particular concern is the pattern of transmission among children, which increases the risk of malabsorption syndrome, a major medical problem. It can lead to complications such as growth retardation, nutritional deficiencies, and weight loss, and severe cases can be fatal (Allain and Buret 2020).

Giardiasis is spread by the fecal–oral route, by which the parasite is transmitted directly or indirectly. Direct transmission includes person-to-person contact or animal-to-animal spread. On the other hand, indirect transmission occurs through contaminated food or water sources, where people ingest the parasite without realizing it (Dixon 2021). Asymptomatic carriers of *Giardia* play a crucial role in the spread of the disease, as they unknowingly transmit the parasite to others, which contributes significantly to its persistence in communities (Ryan et al. 2019). In addition, *Giardia* infections have a zoonotic dimension, especially linked to domestic animals. This zoonotic link underlines that both can harbor and transmit the same genetic strains (Cai et al. 2021).

The therapeutic arsenal against giardiasis is somewhat limited and is mainly based on nitroheterocyclic compounds such as metronidazole, nitazoxanide, and furazolidone. Complementary options include the use of benzimidazole derivatives such as albendazole and mebendazole, along with alternatives such as quinacrine, furazolidone, paromomycin, and nitazoxanide. However, frequent therapeutic failures, often related to inappropriate administration of antiparasitic drugs, pose a major challenge that limits the efficacy of treatment options (Cernikova et al. 2018; Argüello-García et al. 2020). Compounding this problem, these compounds often induce notable side effects, which not only leads to patient noncompliance, but also fosters the emergence of drug-resistant strains (Argüello-García et al. 2020). This underscores the pressing need for innovative therapeutic strategies that not only demonstrate greater accuracy and efficacy, but are also associated with a lower incidence of side effects.

On the other hand, telomeres are the terminal segments located at the ends of linear chromosomes. In vertebrates, telomeric DNA is composed of repetitive TTAGGG sequences, organized and protected by a set of proteins that regulate their biological functions and prevent them from being recognized as DNA double-strand breaks (DSBs), thus preventing the initiation of a DNA damage response (DDR) (Rossiello et al. 2022). The inherent limitations of standard DNA polymerases to fully replicate linear DNA templates, combined with nucleolytic processing during DNA replication, result in the gradual shortening of telomeres (Harley et al. 1990). As telomeres approach a critical length, their ability to bind to sufficient coat proteins decreases, exposing them as DNA ends (de Lange 2009). This exposure activates DDR pathways, triggering the induction of cell cycle inhibitors that prevent cell proliferation (Giardini et al. 2014). Following telomere dysfunction, some cell types may also undergo cell death by apoptosis or autophagy (D’Adda di Fagagna 2008; Nassour et al. 2019). To counteract the natural shortening of telomeres, eukaryotic cells use telomerase, an essential enzyme dedicated to lengthening and preserving these chromosomal ends (Wang et al. 2019).

**Fig. 1:**
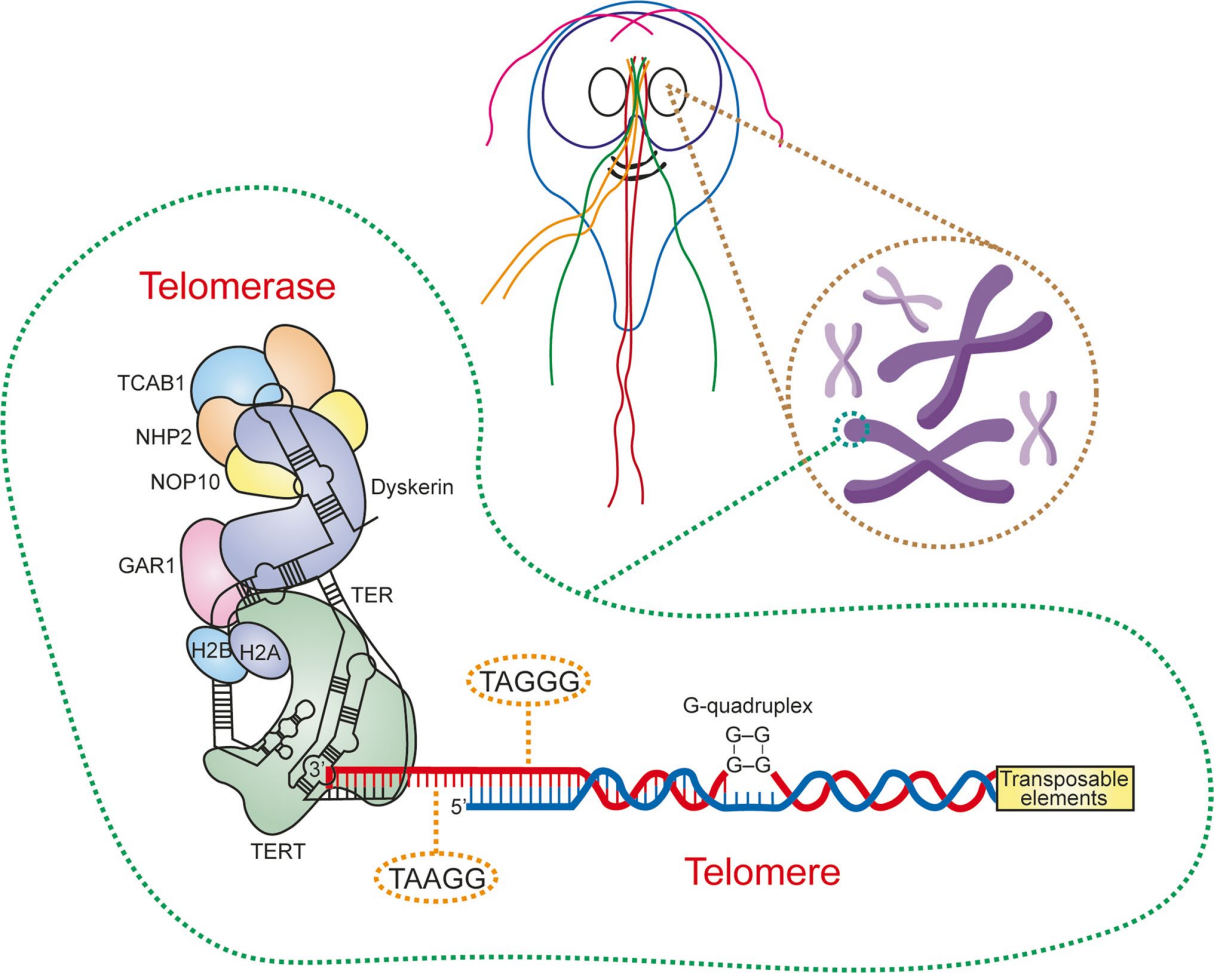
*Giardia* telomeres and telomerase. *Giardia* telomeres are positioned at both ends of all chromatids in its chromosomes. These telomeres are distinguished by TAGGG repeats, with an alternative TAAGG repeat also documented. Telomerase plays a crucial role in elongating the 3′ ends of chromosomes by synthesizing multiple copies of the telomere repeat. This process is facilitated by two specialized components: telomerase reverse transcriptase (TERT) and telomerase RNA (TER)

Despite the identification of *Giardia* telomeres some time ago (Fig. 1) (Le Blancq et al. 1991b; Adam et al. 1991; Uzlíková et al. 2017), our understanding of them, along with the critical role of *Giardia* telomerase in maintaining genomic stability and influencing other cellular processes, remains limited. In this regard, the present study aims to address this knowledge gap by compiling all the currently available information (until 2023) on *Giardia* telomeres and telomerase. Special attention is paid to highlight the distinctive features within this parasite. Furthermore, the potential feasibility of targeting *Giardia* telomeres and/or telomerase as an innovative therapeutic strategy is discussed.

## Telomerase

Telomerase, a DNA-template-independent DNA polymerase, elongates the 3′ ends of chromosomes by synthesizing multiple copies of the telomere repeat. This process is facilitated by its specialized components: telomerase reverse transcriptase (TERT) and telomerase RNA (TER) (Wang et al. 2019). TERT comprises conserved domains, such as the reverse transcriptase (RT, palm and fingers) and the carboxy-terminal element (CTE, thumb), similar to those found in other reverse transcriptases. In addition, it incorporates an telomerase RNA-binding domain (TRBD), collectively forming the TERT ring structure. In addition, TERT has an amino-terminal domain (TEN) connected to the TRBD. Functionally, the TEN domain serves to recruit and enhance telomerase processivity, essentially acting as a “clamp” to extract newly synthesized telomere DNA from the active site and assist in template translocation, one hexamer at a time (Zaug et al. 2008). The TRBD domain establishes a high-affinity binding platform crucial for specific interactions with telomerase RNA components essential for the assembly and function of the holoenzyme (Lai et al. 2001). Within the RT region is the catalytic active site, responsible for the correct alignment of the telomerase RNA template and nucleotide addition during polymerase activity (Hernandez-Sanchez et al. 2016). Finally, the CTE domain plays a functional role in the stabilization of the telomerase-DNA complex, being essential for telomerase-mediated nucleotide addition and processivity (Hossain et al. 2002). Regarding TER, it is a transcript that plays a key role as a template guiding the synthesis of telomeric repeats. Two essential conserved regions facilitate its interaction with TERT: the template/pseudoknot (t/PK) domain, which forms a loop that includes the template and a pseudoknot, and the stem-terminal element (STE), which has a crucial hairpin structure (Theimer and Feigon 2006; Zhang et al. 2011).

## *Giardia* telomeres

Within each *Giardia* trophozoite are two nuclei, each of which harbors five very small chromosomes ranging from ~ 0.8 to 2.4 µm. These chromosomes have a single centromeric locus and are capped by telomeric repeats (Tůmová et al. 2015). In addition, there are reports of secondary or accessory chromosomes of variable length in *Giardia*, which contain sets of ribosomal DNA repeats (rDNA) (Upcroft et al. 2010). Notably, high levels of chromosomal instability and frequent mitotic segregation errors have been observed in *Giardia*, contributing to phenomena such as whole chromosome aneuploidy, unequal gene distribution, and remarkable genomic divergence between the two nuclei of the same cell (Tůmová et al. 2016). The terminal regions of each *Giardia* chromosome are characterized by a subtelomeric region that culminates in the telomere gene unit (TGU). This segment has conserved arrangements, with a cysteine-rich protein gene (or variable surface protein gene, VSP), a protein kinase gene (gPK), and an ankyrin gene (ANK) (Upcroft et al. 2010). Following this organized sequence, interspersed with hypervariable segments containing transposable elements, are ribosomal RNA (rRNA) genes and telomere terminal repeats (Prabhu et al. 2007). Towards the end of the ribosomal RNA (rRNA) gene tandem, a truncated form of the 18S subunit appears and is abruptly interrupted by the telomere sequence (Upcroft et al. 1997; Lagunas-Rangel 2023).

The telomeres of *Giardia* are located at both chromatid ends of all its chromosomes (Adam et al. 1991; Tůmová et al. 2015). Employing the terminal restriction fragment (TRF) method, *Giardia* telomeres exhibit lengths ranging from 500 bp to 2.5 kb in most cell lines, with an absence of long interstitial telomeric sequences (Uzlíková et al. 2017). These are characterized by TAGGG repeats, and an alternative repeat, TAAGG, has also been documented (Le Blancq et al. 1991a; Adam et al. 1991). This sequence closely resembles the predominant telomeric repeat in humans and other vertebrates, TTAGGG, and is also observed in most representatives of Excavata in which telomeric sequences have been studied (Fulnečková et al. 2013). The identification of variant sequences suggests that *Giardia* telomeres may be composed of diverse telomeric repeats synthesized by telomerase. This hypothesis is reinforced by the observation of an irregular staircase pattern in the telomeric repeat amplification protocol (TRAP) assay (Uzlíková et al. 2017). The occurrence of multiple telomere variants within the telomere is not uncommon, as imperfect synthesis of telomeric repeats has been identified in other eukaryotes, including plants from the order Asparagales (Sýkorová et al. 2003). In this context, a common error for many telomerases is T/G slippage, characterized by the addition of one or more Ts or Gs to a synthesized telomere repeat (Fitzgerald et al. 2001).

Telomere detection in *Giardia* covers both trophozoite and cyst phases, as well as all phases of the trophozoite cell cycle (Adam et al. 1991; Carpenter et al. 2012; Uzlíková et al. 2017), revealing distinct patterns in the distribution of telomeric foci. In particular, clustering is more pronounced during the G2 and mitosis phases compared to the G1 phase (Uzlíková et al. 2017). Furthermore, in the G1 phase, the foci were clustered at opposite nuclear poles, indicating their tendency to cluster in regions close to the nuclear periphery (Carpenter et al. 2012; Uzlíková et al. 2017). It is noteworthy that, unlike several mammalian cells in which G1 represents the longest phase of the cell cycle, *Giardia* trophozoites spend most of their cell cycle in the G2 phase (Bernander et al. 2001). In this sense, clustering of telomeric foci has been proposed as a mechanism through which nuclei could undergo recombination (Carpenter et al. 2012). Clustering of chromosomal ends has also been documented in *Trypanosoma* and *Plasmodium* (parasites not phylogenetically closer to *Giardia*) and has been proposed to be involved in shuffling parasite surface antigens (Chung et al. 1990; Scherf 2001).

## *Giardia* telomerase

**Fig. 2:**
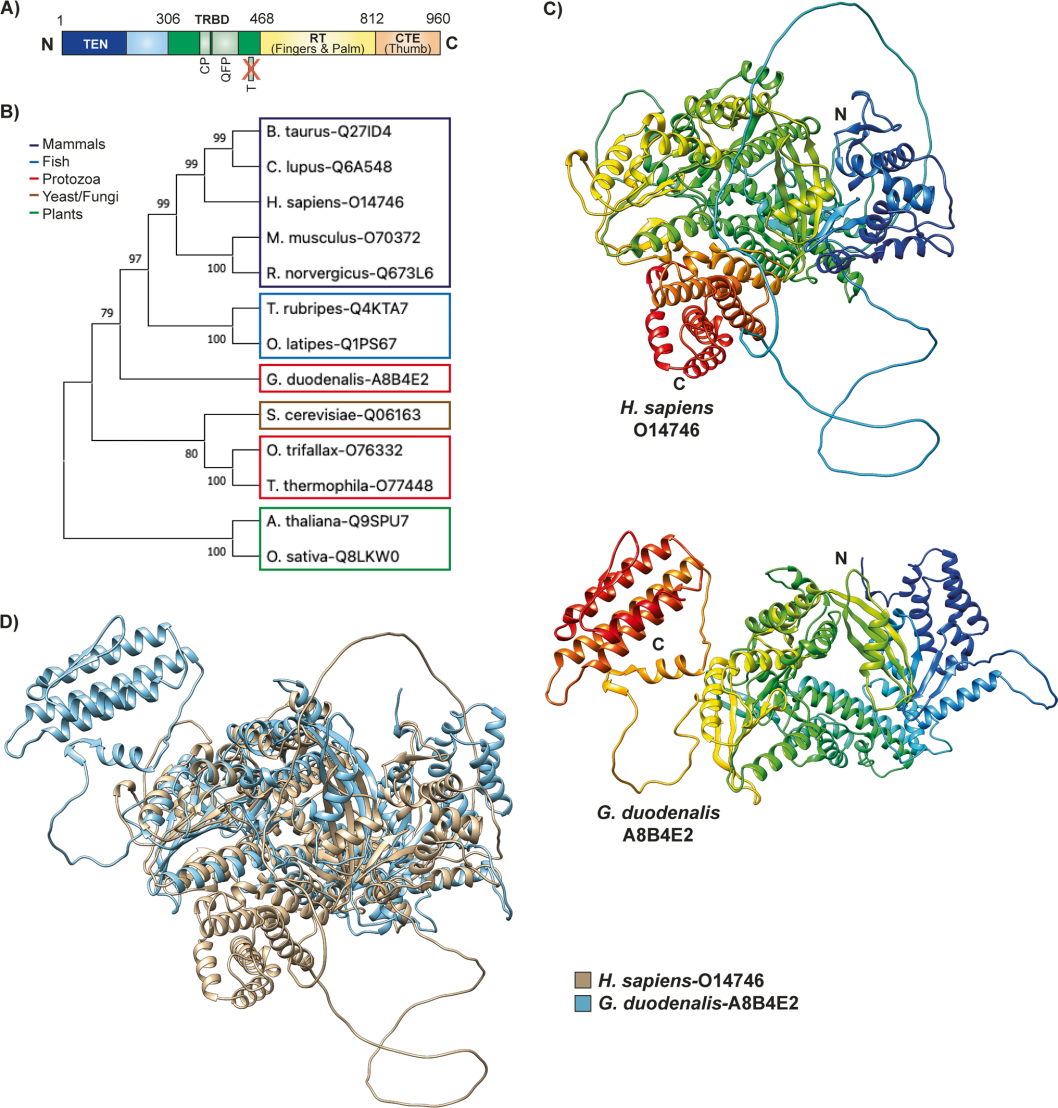
*Giardia* telomerase reverse transcriptase (TERT). **A** *Giardia* TERT exhibits four distinctive domains: the amino-terminal domain (TEN), the telomerase RNA-binding domain (TRBD), the reverse transcriptase (RT) domain, and the carboxy-terminal element (CTE) domain. Notably, the TRBD domain lacks the telomerase-specific “T” motif. **B** Phylogenetic tree constructed from TERT ortholog sequences of selected species, including *Giardia*. Distances between species were determined using the maximum likelihood algorithm, showing next to the branches the percentage of replicates in which the associated taxa clustered in the bootstrap test (100 replicates). Species are classified into different groups by colored boxes: mammals (purple), fish (blue), protozoa (red), yeasts/fungi (brown), and plants (green). **C** Three-dimensional structure of human and *Giardia* TERT. The structures were predicted using AlphaFold (Varadi et al. 2022) and based on sequence information available from UniProt KD (Bateman et al. 2021). **D** Superposition of the structure of *Giardia* TERT and the human homologue. The TRBD domain and the RT domain are the most conserved

Encoded by the *Giardia* TERT gene (GL50803_16225), which consists of a single exon, *Giardia* TERT protein consists of 960 amino acids, with a molecular weight of approximately 110 KDa and an isoelectric point of 9.7 (Fig. 2A). Despite its thermolability, a characteristic shared with other telomerases (Uzlíková et al. 2017), *Giardia* TERT is considered a relatively stable protein with an estimated half-life of more than 10 h. Furthermore, *Giardia* TERT has a higher percentage of serines (10.7%) compared to human (6.6%), mouse (8.7%), and yeast (7.8%) TERT. This disparity in serine content could potentially serve as a molecular defense mechanism against oxidative stress.

Phylogenetically, *Giardia* TERT branched off from the evolutionary lineages of mammalian and yeast counterparts several years ago, marking a significant separation from these organisms (Fig. 2B). Furthermore, it also distancing itself from other non-parasitic protozoa. *Giardia* TERT has 14.75% identity and 31.84% similarity to its yeast counterpart, as well as 15.81% identity and 30.11% similarity to the corresponding human protein. Despite the relatively low sequence conservation, the structural integrity of *Giardia* TERT remains preserved (Fig. 2C).

Structurally, it adheres to the classical telomerase framework, presenting the four distinctive domains: the TEN domain, the TRBD domain, the RT domain, and the CTE domain (Malik et al. 2000). The regions showing the highest degree of conservation are located within the TRBD domain and the RT domain (Fig. 2D). Notably, *Giardia* TRBD domain is distinguished by lacking the telomerase-specific “T” motif, which could reduce its processivity (Malik et al. 2000; Gramatges et al. 2013). Despite this, *Giardia* exhibits constant telomerase activity, as confirmed by a TRAP assay (Uzlíková et al. 2017). This activity leads to the synthesis of the characteristic *Giardia* telomeric sequence TAGGG (Adam et al. 1991), as well as other repeat variants such as TAAGG and TAAGGG (Uzlíková et al. 2017).

**Fig. 3:**
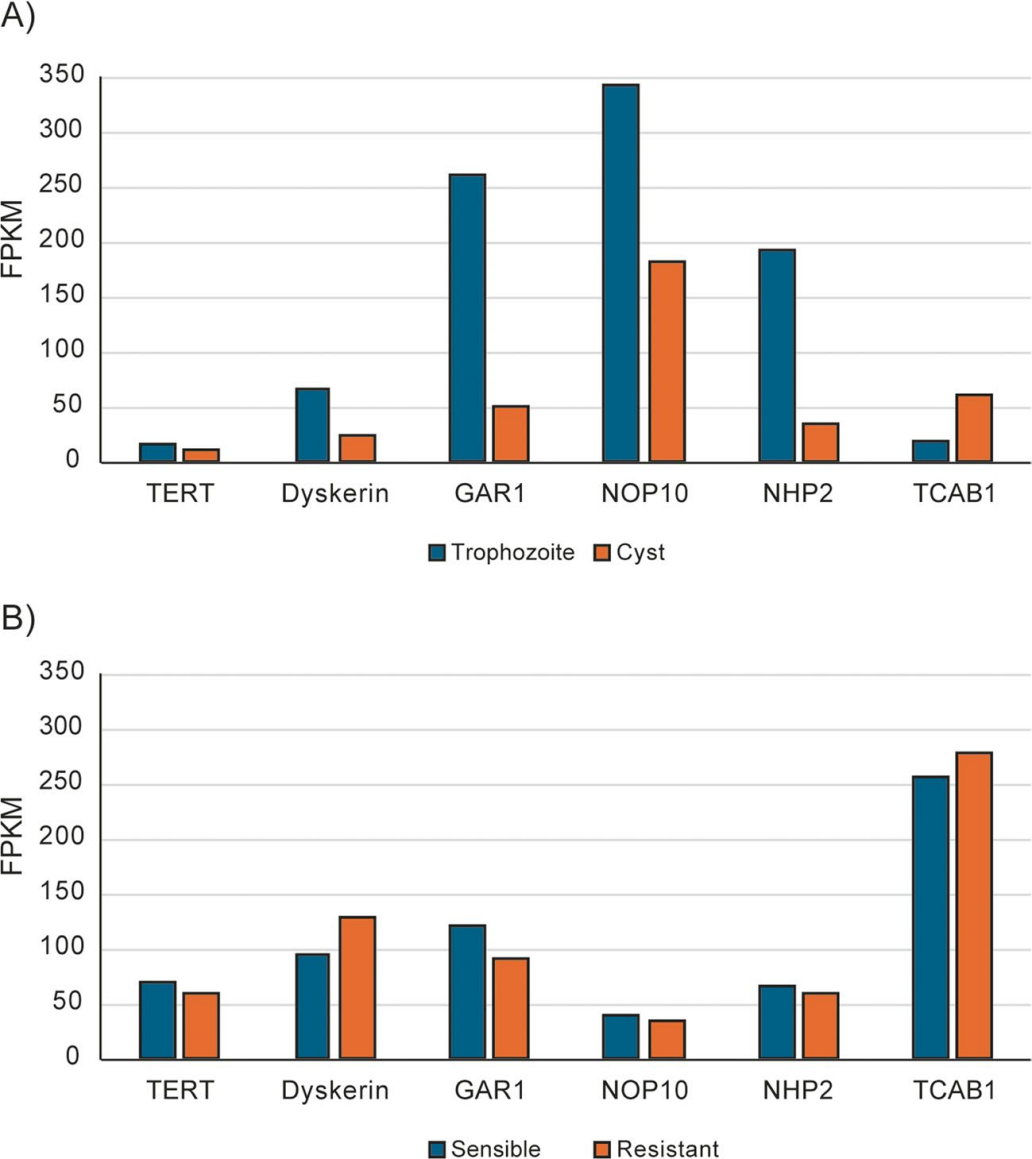
Transcriptional changes of *Giardia* telomerase components during the different processes. **A** Changes during encystation. Graph created using data from Einarsson et al. 2016b. **B** Changes that occur during the development of *Giardia* resistance to metronidazole. Graph created using data from Ansell et al. (2017). FPKM, fragments per kilobase million

Additional components of human telomerase that may potentially also be present in *Giardia* include dyskerin (GL50803_16311), GAR1 (GL50803_8794), NOP10 (GL50803_8242), NHP2 (GL50803_13926), and TCAB1 (GL50803_11953). However, experimental validation of this is needed, as well as confirmation of the functional similarity of these *Giardia* components to their human counterparts. Notably, during the transformation of the trophozoite into a cyst, the expression of most *Giardia* telomerase components appears to be markedly reduced (Fig. 3A). However, these components could be reactivated during de-cystification. This suggests that *Giardia* telomerase activity may be reduced during the cyst phase. Interestingly, no consistent changes in these components were observed when comparing metronidazole-resistant strains with sensitive strains (Fig. 3B).

Telomerase is localized in the nuclei of both *Giardia* trophozoites and cysts (Uzlíková et al. 2017). The persistent telomerase activity observed in *Giardia* populations is a characteristic shared with other highly proliferative unicellular eukaryotic pathogens. This activity ensures the maintenance of a constant telomere length, which facilitates sustained propagation within its host (Dey and Chakrabarti 2018). However, *Giardia* telomerase activity appears to be regulated, as evidenced by the consistent pattern observed in the telomeric repeat amplification protocol assay, even in cases of overexpression (Uzlíková et al. 2017). In addition, the interaction of the *Giardia* zinc finger domain (ZFD) protein (GL50803_20802) with the TRBD domain of TERT has been described. Surprisingly, the silencing of this protein is related to a decrease in the growth of parasitic cells, attributed to a decrease in telomerase activity and, consequently, to a reduction in telomere length. Thus, it is suggested that the *Giardia* ZFD protein plays a role as a positive regulator of TERT activity (Zheng et al. 2019). Likewise, an interaction between *Giardia* replication factor 1 subunit C (RFC1) (GL50803_15392) and *Giardia* TRBD has been described, with a consequent positive effect on TERT activity. Thus, silencing of RFC1 has been associated with a reduction in *Giardia* telomerase activity (Li et al. 2020). This is in contrast to what has been reported with the human homologue of this protein that has telomerase inhibitory activities (Uchiumi et al. 1999).

## Other mechanisms in *Giardia* for telomere maintenance

Besides telomerase activity, *Giardia* exhibits additional mechanisms for telomere maintenance. Some studies have reported that the telomeric sequence TAGGG of *Giardia* forms modified G-quadruplex structures that possibly collaborate in telomere maintenance (Hu et al. 2009; Bansal and Kukreti 2020). Indeed, based on this, a potential mechanism for telomere-telomere association has been suggested, involving G-overhangs at specific stages of the cell cycle. This mechanism could contribute to the connection of the 3′ protrusions of two sister chromatids, forming a V-shaped structure, positioned opposite each other during anaphase. This arrangement allows end-to-end (antiparallel) association of the chromatids across their telomeres (Bansal and Kukreti 2020).

On the other hand, the discovery of transposable elements (e.g., LINE-like elements) linked to telomeric repeats in *Giardia* has raised speculation about the possible role of retrotransposons in telomere maintenance (Arkhipova and Morrison 2001). The *Giardia* genome harbors three retrotransposons, with two of them (GilT and GilM) positioned within the telomeric region (Arkhipova and Morrison 2001). Subterminal positioning reduces the likelihood of transposable elements (either GilM or GilT elements) interfering with gene function (Pardue et al. 2001). Moreover, these elements are strategically oriented to guide reverse transcription towards the chromosome end, suggesting a possible redundancy with telomerase activities. This phenomenon is reminiscent of that in *Drosophila*, where the absence of telomerase is compensated for by the TART retroposon and its dependent HeT-A (Biessmann et al. 1992). It is worth mentioning that a substantial amount of small endo-RNAs (sRNAs), RNAs with sizes of less than 40 nucleotides, are generated from telomeric *Giardia* retrotransposons in active transcription. These sRNAs target mRNAs in trans and are involved in various processes such as encystation, stress responses, and retrotransposon silencing (Ullu et al. 2005; Liao et al. 2014).

Remarkably, no component of the shelterin complex, responsible for protecting telomeric DNA against unwanted degradation and end-to-end fusion events, has been identified in *Giardia*.

## Targeting *Giardia* telomeres and telomerase

In the field of cancer treatment, telomerase-targeted therapies have historically shown great promise (Gao and Pickett 2022). This concept could be extended to other undesirable cells, such as parasites, in this case *Giardia*. Indeed, the common action of anticancer and antiparasitic drugs has been described previously (Dorosti et al. 2014). Given the pivotal role of telomeres and telomerase in *Giardia*, targeting these components emerges as a viable and novel therapeutic strategy against giardiasis. This notion is reinforced by observations indicating that a decrease in telomerase activity and/or a reduction in telomere length leads to a decrease in the growth of parasitic cells (Zheng et al. 2019; Li et al. 2020). This strategy could consist of targeting the *Giardia* TERT and/or the proteins that positively regulate its activity, evaluating both the direct antiparasitic effects and the possible synergy with currently used antigiardiasis drugs. A starting point could be to take as a basis BIBR1532, the most successful small molecule inhibitor of human TERT. BIBR1532 binds to a non-catalytic site of human TERT, inhibiting telomerase activity with non-competitive kinetics (Bryan et al. 2015). This compound interacts with the hydrophobic pocket of the thumb domain of telomerase, preventing the translocation necessary for the processive addition of telomeric repeats (Pascolo et al. 2002; Bryan et al. 2015). Designing similar compounds specifically tailored to *Giardia* TERT and performing in vitro and in vivo studies would be the first steps. Sequence divergence between *Giardia* TERT and human TERT offers the opportunity to identify inhibitory compounds that act specifically on the parasite. This specificity holds promise for minimizing potential host side effects, improving the efficacy and safety of treatment options.

This strategy, however, has important limitations, such as the long interval from telomerase inhibition to the critical point of telomere shortening required for a cytotoxic effect. In addition, the designed compounds must show specificity for *Giardia* telomerase to avoid potential toxicity towards highly proliferative tissue compartments in human hosts. Also, the development of new antiparasitic drugs faces limitations, mainly due to the significant investment required for research in molecular parasitology and the infrastructure needed for the development and screening of drug candidates. This often discourages pharmaceutical companies from undertaking the development of such drugs (Woods and Williams 2007).

## Conclusions

Existing knowledge of *Giardia* telomeres and telomerase remains quite limited, underscoring the need for further research to deepen our understanding. Indeed, one of the main objectives of this paper is to promote and inspire future research efforts dedicated to this often overlooked topic. *Giardia* telomeres possess distinctive characteristics, which are also reflected in the unique nature of its telomerase enzyme, and both play critical roles in this parasitic organism. Therefore, exploring *Giardia* telomerase as a potential therapeutic target is emerging as a viable strategy, especially in view of the increasing cases of drug-resistant strains. However, it is essential to thoroughly analyze the limitations of this strategy. Here, it is highlighted that the *Giardia* telomeric sequence, TAGGG, is accompanied by other repeat variants, both capable of forming G-quadruplex structures. In addition, the presence of transposable elements linked to the telomeric repeats is highlighted, suggesting a possible association with telomere maintenance. Similarly, it is highlighted that *Giardia* telomerase demonstrates continuous activity, even without the telomerase-specific “T” motif, and that its activity is regulated by other proteins. Much remains to be explored in this area. For example, the sequence and structure of *Giardia* TER remain to be unraveled. Furthermore, future studies should deepen the identification of TERT-interacting proteins, elucidating their role in the regulation of telomerase activity in *Giardia*. Expanding our understanding of the mechanisms of telomere protection in *Giardia* is very promising. Moreover, the search for specific inhibitors adapted to this context could not only improve our understanding, but also pave the way for possible therapeutic applications. Inhibitors directed against *Giardia* TERT could be used alone or together with other antiparasitic drugs, potentially synergizing or amplifying their cytotoxic effects against the parasite.

## Data Availability

No datasets were generated or analysed during the current study.
